# Supplementary material for: Clinical Implications of Neurological Comorbidities and Complications in ICU Patients with COVID-19
Source: J Clin Med. 2021 May 25;10(11):2281. doi: 10.3390/jcm10112281 (PMC8197394; doi:10.3390/jcm10112281)
Supplement: Supplementary file 1 [file jcm-10-02281-s001.zip › jcm-1196199-supplementary.pdf]

Table S1. Independent predictor associated with ICU mortality in multiple logistic regression analysis using SOFA score.

	**OR**	**95% C.I.**	**p-value**
Age	1.01	0.947—1.07	0.81
Male	4.55	0.75—27.60	0.10
SOFA score	1.74	1.21—2.51	0.003
New-onset neurologic complications	6.27	1.06—37.22	0.044

Nagelkerke R2 = 0.638

Hosmer-Lemeshow Chi-square test = 4.404, df=8, p-value=0.819

	OR	95% C.I.	p-value
Age	1.01	0.95—1.08	0.78
Male	5.34	0.75—38.00	0.10
SOFA score	1.76	1.21—2.56	0.003
Preexisting neurological comorbidities	0.65	0.09—4.55	0.66
New-onset neurologic complications	7.45	1.05—53.06	0.045

Nagelkerke R2 = 0.641; Hosmer-Lemeshow Chi-square test = 3.991, df = 8, *p*-value = 0.858; CI, confidence interval; ICU, intensive care unit; OR, odds ratio; SOFA, Sequential Organ Failure Assessment.

Table S2. Baseline characteristics of 52 intensive care unit patients infected with COVID-19 according to delirium.

	**All ICU patients N=52**	**No delirium N=32 (61.5%)**	**Delirium N=20 (38.5%)**	**p value**
Characteristics				
Age, median (IQR), year	73 (57 - 81)	70 (54 - 79)	77 (60 - 85)	0.135
Sex, male (%)	27 (51.9)	16 (50.0)	11 (55.0)	0.017
Underlying medical conditions (%)				
Hypertension	29 (55.8)	18 (56.3)	11 (55.0)	0.930
Diabetes mellitus	24 (46.2)	17 (53.1)	7 (35.0)	0.202
Dyslipidemia	5 (9.5)	1 (3.1)	4 (20.0)	0.066
Cardiac disease	9 (17.3)	3 (9.4)	6 (30.0)	0.071
Malignancy	6 (11.5)	3 (9.4)	3 (15.0)	0.664
Chronic liver disease	2 (3.8)	1 (3.1)	1 (5.0)	1
Chronic kidney disease	6 (11.5)	1 (3.1)	5 (25.0)	0.026
Scoring system, median (IQR)				
SOFA score	4 (2 - 6)	3 (1 - 5)	5 (3 - 7)	0.0.66
APACHE II score	11 (7 - 21)	10 (7 – 21)	12 (9 – 21)	0.202
Preexisting neurologic comorbidities (%)	19 (36.5)	6 (18.8)	13 (65.0)	0.001
Neurodegenerative disease	7 (13.5)	2 (6.3)	5 (25.0)	0.092
Alzheimer disease and other dementias	4 (7.7)	0 (0)	4 (20.0)	0.018
PD and PD-related disorders	2 (3.8)	1 (3.1)	1 (5.0)	1
Motor neuron disease	2 (3.8)	1 (3.1)	1 (5.0)	1
Cerebrovascular disease	7 (13.5)	3 (9.4)	4 (20.0)	0.408
Other neurologic disease	5 (9.6)	1 (3.1)	4 (20.0)	0.066
Other medical complications (%)				
Acute respiratory distress syndrome	21 (40.4)	8 (25.0)	13 (65.0)	0.004
Acute kidney injury	13 (25)	7 (21.9)	6 (30.0)	0.510
Septic shock	22 (42.3)	11 (34.4)	11 (55.0)	0.143
Treatment Timeline, median (IQR), days - n/N.				
length of ICU stays	22 (9 - 32)	20 (9 - 29)	25 (9 - 40)	0.387
Invasive ventilator days, 23/52	18 (3 - 29)	7 (2 - 29) 10/32	18 (7 - 39) 13/20	0.320
Onset-to-ICU admission, 39/52	8 (6 - 11)	8 (7 - 11) 25/32	8 (7 - 10) 14/20	0.918
Prognosis - n/N. (%)				
Transfer out of ICU	33/52 (63.5)	24/32 (75.0)	9/20 (45.0)	0.029
ICU death	19/52 (36.5)	8/32 (25.0)	11/20 (55.0)	

COVID-19, coronavirus disease 2019; IQR, interquartile range; SOFA, Sequential Organ Failure; APACHE, Acute Physiology and Chronic Health Evaluation; ICU, intensive care unit; PD, Parkinson disease. Data are median (interquartile range), n (%), or n/N (%), where N is the total number of patients with available data. *p* values comparing neurological comorbidities and no neurological comorbidities are from $\chi 2$ test, Fisher' exact test, or Mann-Whitney U test.
